# Epigenetic alterations of *miR-155 *and global DNA methylation as potential mediators of ochratoxin A cytotoxicity and carcinogenicity in human lung fibroblasts

**DOI:** 10.1007/s11356-023-31283-0

**Published:** 2023-12-20

**Authors:** Taghrid G. Kharboush, Inas A. Ahmed, Amina A. Farag, Tayseir Kharboush, Alaa El-Din H. Sayed, Amal M. Abdel-Kareim, Mohammed Al mohaini, Hend Attia, Refaat A. Eid, Mohamed Samir A. Zaki, Al-Shaimaa M. Al-Tabbakh

**Affiliations:** 1 Department of Medical Microbiology and Immunology, Faculty of Medicine, Benha University, Benha, 13518 Egypt; 2 Department of Medical Biochemistry and Molecular Biology, Faculty of Medicine, Benha University, Benha, 13518 Egypt; 3 Central Laboratory for Research, Faculty of Medicine, Benha University, Benha, 13518 Egypt; 4 Department of Forensic Medicine & Clinical Toxicology, Faculty of Medicine, Benha University, Benha, 13518 Egypt; 5 Department of Pharmacology and Therapeutics, Faculty of Medicine, Benha University, Benha, 13518 Egypt; 6 Department of Zoology, Faculty of Science, Assiut University, Asyut, 71516 Egypt; 7 Molecular Biology Research & Studies Institute, Assiut University, Asyut, 71516 Egypt; 8 Department of Zoology, Faculty of Science, Benha University, Benha, 13518 Egypt; 9 Basic Sciences Department, College of Applied Medical Sciences, King Saud Bin Abdulaziz University for Health Sciences, 31982 Alahsa, Saudi Arabia; 10 King Abdullah International Medical Research Center, 31982 Alahsa, Saudi Arabia; 11 Clinical and Chemical Pathology, School of Medicine, Newgiza University (NGU), Giza, Egypt; 12 Department of Pathology, College of Medicine, King Khalid University, P.O. Box 62529, Abha, Saudi Arabia; 13 Department of Anatomy, College of Medicine, King Khalid University, P.O. Box 62529, Abha, Saudi Arabia

**Keywords:** Ochratoxin A 1; Epigenetics 2; miRNA-155 3, DNA methylation 4, Cytotoxicity 5 lung cells

## Abstract

Ochratoxin A (OTA) is a well-known mycotoxin that adversely affects different human cells. Inhalational exposure to OTA and subsequent pulmonary diseases have been previously reported, yet its potential carcinogenicity and underlying molecular mechanisms have not been fully elucidated. This study aimed to evaluate the OTA-induced cytotoxicity and the epigenetic changes underlying its potential carcinogenicity in fetal lung fibroblast (WI-38) cells. OTA cytotoxicity was assessed by MTT assay; RT-qPCR was used to determine the expression of *BAX*, *BCL-2*, *TP53*, and *miR-155*, while ELISA was used for measuring 5-methyl cytosine percentage to assess global DNA methylation in OTA-treated versus control cells. WI-38 cells demonstrated sensitivity to OTA with IC50 at 22.38 μM. Though *BAX* and *Bcl-2* were downregulated, with low *BAX*/*BCL-2* ratio, and *TP53* was upregulated, their fold changes showed decline trend with increasing OTA concentration. A significant dose-dependent miR-155 upregulation was observed, with dynamic time-related decline. Using subtoxic OTA concentrations, a significant global DNA hypermethylation with significant dose-dependent and dynamic alterations was identified. Global DNA hypermethylation and miR-155 upregulation are epigenetic mechanisms that mediate OTA toxicity on WI-38 cells. *BAX* downregulation, reduced *BAX*/*BCL-2* ratio together with *miR-155 *upregulation indicated either the inhibition of *TP53*-dependent apoptosis or a tissue specific response to OTA exposure. The aforementioned OTA-induced variations present a new molecular evidence of OTA cytotoxicity and possible carcinogenicity in lung fibroblast cells.

**Graphical Abstract:**

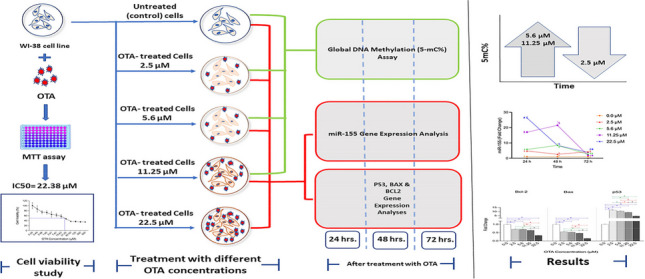

## Introduction

Ochratoxin A (OTA) is a mycotoxin that was first isolated from *Aspergillus ochraceus* (*A. ochraceus*) in 1965 and found to jeopardize public health by contaminating the food chain worldwide (van der Merwe et al. 1965; Zhu et al. 2017). It is produced by different fungi related to the following genera: *Penicillium* (*P.*) and* Aspergillus* (*A.*), e.g., *A. carbonarius*, *A. ochraceus* and* P. verrucosum*. In addition to its nephrotoxic effects, hepatotoxic, teratogenic, neurotoxic, and immune-toxic activities have also been reported (Awuchi et al. 2021; Chain et al. 2020).

Inhalational exposure to OTA was identified in many studies, and airborne dust, bioaerosols besides the conidia were established as possible sources of exposure to OTA (Warensjö Lemming et al. 2020). OTA-producing airborne *Aspergilli* were also isolated from the indoor air collected at post-flood locations. The impact of mycotoxins on adverse health effects after inhalational exposure remains debatable (Jakšić et al. 2020).

Various cellular hazardous consequences are linked to OTA exposure such as arrest of the cell cycle, DNA damage, disruption of the protein synthesis, necrosis, triggering apoptosis, and degradation of the chromosomes (Costa et al. 2016; Marin-Kuan et al. 2011; Zhu et al. 2017). Furthermore, OTA had been classified as a possible human group 2B carcinogen, by the International Agency for Research on Cancer (IARC) (Zhu et al. 2017). The proto-oncogene, B cell lymphoma (*BCL-2*), and tumor suppressor gene, tumor protein p53 (*TP53*), are among the earliest recognized cancer regulating genes (Lane and Crawford 1979; Linzer and Levine 1979), which show differential significance during carcinogenesis according to the tumorigenic contexts (Hemann and Lowe 2006). Previous research had shown that genotoxic carcinogens activate the *TP53* gene products that trigger the production of genes such as *BCL-2*, in response to DNA damage (Ellinger-Ziegelbauer et al. 2004; van Delft et al. 2004). Likewise, the transcriptional activation of p53 target genes was deliberated as an early and global indicator of genotoxic stress (Zerdoumi et al. 2015).

Altered DNA methylation is an important epigenetic mechanism that is related to genomic instability and tumor suppressor genes silencing. Other epigenetic modulators include the small non-coding RNAs (miRNAs). Surprisingly, miRNA expression can be affected by other epigenetic modulators. Changes of miRNAs expression had been shown to link the exposure to certain environmental toxins with their pathological effects, such as the onset and progression of cancer (Zerdoumi et al. 2015). MiR-155 acts as an oncomiR in T cell lymphoma and in numerous solid neoplasms (Gironella et al. 2007; Yu et al. 2017). miR-155 was abnormally upregulated in the serum and tissues of patients with cancer lung. Furthermore, patients with high miR-155 expression in their lung were reported to have poor prognosis and short lifespan (Liu et al. 2017; Zhu et al. 2018). Following OTA treatment, significant increase in miR-155-5p was detected in the kidney tissue of animals (Yang et al. 2023). MiR-155-5p was also shown to be the most increased miRNA by next-generation sequencing (NGS) in Caco-2 cells and mice colon tissues after exposure to OTA (Rhee et al. 2023). Additionally, miR-155 plays a pivotal role in regulating *TP53INP1* gene expression (Ng et al. 2011; Zhang et al. 2014). *TP53INP1* gene is a tumor suppressor that triggers apoptosis and cell growth arrest by modifying the transcriptional activity of p53 (Shahbazi et al. 2013). The study of mycotoxin epigenetics is important because it creates a framework for cancer epigenetic studies. The kidney and liver are almost the only organs where the OTA-induced epigenetic alterations have been studied. Therefore, more precise changes to the epigenetic pathways of OTA-induced toxicity should be investigated (Zhu et al. 2017).

A limited number of research studies were conducted to evaluate the perilous effects of OTA on lung-derived cells. It is still not fully understood what molecular mechanisms underlie such effects. Thus, it is crucial to ascertain whether or not epigenetic mechanisms are responsible for OTA-induced cytotoxicity and possible carcinogenicity in cells obtained from human lungs. Hence, we aimed to assess the altered expression of apoptosis regulatory genes (*TP53*,* BAX*,* BCL-2*) and some cancer-related epigenetic markers (oncomiR-155 and global DNA methylation) as potential evidence of OTA-induced cytotoxicity and possible carcinogenicity in fetal lung fibroblast (WI-38) cells.

## Materials and methods

### Experimental design (Fig. 1)

**Fig. 1:**
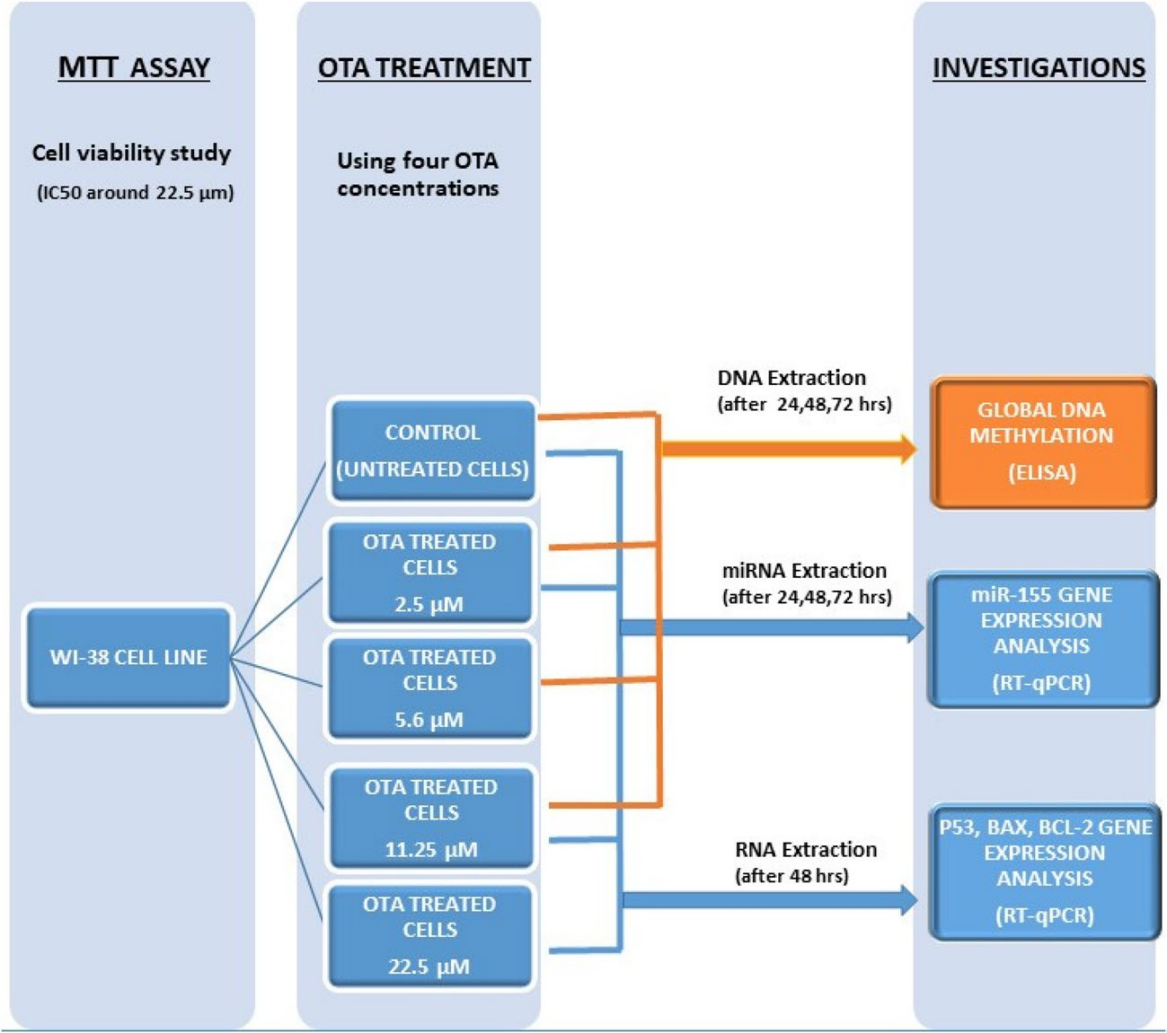
Diagram illustrating the experimental design and workflow throughout the study

#### Chemicals

The following is a list of all of the chemicals and their manufacturers used in this study: Dulbecco’s modified Eagle’s medium (DMEM), fetal bovine serum (FBS) (GIBCO, ThermoFisher Scientific, Waltham, MA, USA), trypsin–EDTA (GIBCO), phosphate-buffered saline (PBS, pH = 7.2 ± 0.2) (Adwia Pharmaceuticals, El Sharkeya, Egypt), mixture of 100 IU/ml penicillin, and 100 mg/ml streptomycin, (Sigma-Aldrich, MO, USA). Ochratoxin A (OTA), catalogue number O1877, purity ≥ 98%, HPLC grade was purchased from (Sigma-Aldrich, USA), MTT reagent (3-(4,5-dimethylthiazol-2-yl)-2,5-diphenyltetrazolium bromide), 0.01 M solution dimethylsulfoxide (DMSO) (ICI-UK).

#### Cell culture and treatment

WI-38 cells American Type Culture Collection (ATCC#CCL-75) was kindly supplied from the Tissue Culture Department, for production of Vaccines, Sera, and Drugs (VACSERA, Giza, Egypt). Cells were grown according to manufacturing protocol in DMEM supplemented with 10% FBS, a mixture of 100 IU/ml penicillin, and 100 μg/ml streptomycin, 1 mM pyruvate, and 1 g/L glucose, Gibco at 37 °C in 5% CO_2_ incubator (Jouan SA, Saint-herblain, Pays de la Loire, France).

#### Cell viability and IC50 evaluation (MTT assay)

Cells were plated at a concentration of 2 × 10^5^ cell/ml in DMEM growth medium, distributed as 100 μL in 96-well plate (TPP-Swiss) followed by incubation at 37 °C till confluency is reached. The growth medium was decanted and fresh medium containing twofold serially diluted OTA to pre-cultured plate. The treatment concentrations of OTA were made up of serum-free basal medium (DMEM) with OTA at several concentrations measured in μM/ml (250, 125, 62.5, 31.25, 15.63, 7.81, 3.91, 1.95, 0.98, 0.49, and 0.24). Untreated WI-38 cells were used as control.

Forty-eight hours later, dead cells were washed out using PBS, and 50 μl of MTT stain stock solution (0.5 mg/ml) was added to each well. The supernatant was discarded after 4 h of incubation at 37 °C. Then, 50 μl/well of DMSO (ICI-UK) were added to solubilize the purple formazan. Formazan is known to be produced in viable cells by the mitochondrial enzyme succinate dehydrogenase. The plate was incubated at 37 °C for 30 min in the dark, and the absorbance was measured at a wavelength of 570 nm using microplate reader (ELx-800, Bio-Tek Instruments, Inc., Winooski, VT, USA). The following formula was used to calculate the percentage (%) of the cell viability: viability % = mean OD of test dilution (in presence of OTA) × 100/mean OD of negative control wells. From the MTT assay results, the half-maximal inhibitory concentration (IC50) of OTA was calculated as the concentration inducing 50% loss of cell viability. The IC50 value was determined using GraphPad Prism software (v.6, GraphPad Software, La Jolla, CA, USA) (Said et al. 2022).

#### RNA extraction

Cells were harvested at 24, 48, and 72 h after OTA treatment for RNAs extraction. Total RNA, including miRNA, was extracted from negative control cells and OTA-treated WI-38 cells at the following concentrations 22.5 μM, 11.25 μM, 5.6 μM, and 2.5 μM. At first, total RNA was extracted using miRNeasy Mini Kit (Qiagen, Hilden, Germany), followed by separation of the miRNA- and mRNA-enriched fractions using the RNeasy MinElute Cleanup Kit (Qiagen, Hilden, Germany), according to the manufacturers’ protocol (Beasley et al. 2022). The concentration and purity of the extracted RNA was evaluated using NanoDrop One spectrophotometer (Thermo Fisher Scientific, MA, USA).

### Reverse transcription quantitative polymerase chain reaction

#### Apoptosis regulatory genes

A concentration of 500 ng of freshly extracted mRNA at 48 h, was immediately used for cDNA synthesis by T-100 thermal cycler (Bio-Rad, CA, USA). The high-capacity cDNA reverse transcriptase Kit (Thermo Fisher Scientific, MA, USA) was used following the manufacturer’s instructions and then cDNA samples were immediately kept at − 20°C till further PCR amplification.

The obtained cDNA was subsequently amplified using Quantitect Syber green Master mix (Qiagen, Hilden, Germany), by StepOnePlus Real-Time PCR System (Applied Biosystems, Thermo Fischer Scientific, USA). The PCR program was adjusted as follows: enzyme activation step, 10 min at 95 °C. The amplification step, 40 cycles of 15 s at 95 °C, 20 s at 55 °C, and 30 s at 72 °C. The following primer sequences of apoptosis-related genes and β-actin (Ramadan et al. 2019) were used as follows:

- *BAX*: F 5'-ATG GAC GGG TCC GGG GAG CA -3', R 5'- CCC AGT TGA AGT TGC CGT CA-3'
- *BCL-2:* F 5'- GTG AAC TGG GGG AGG ATT GT -3', R 5'- GGA GAA ATC AAA CAG AGG CC -3'
- *TP53*: F 5'- TCA GAT CCT AGC GTC GAG CCC-3', R 5'- GGG TGT GGA ATC AAC CCA CAG-3'
- Β-actin: F 5’- CTG TCT GGC GGC ACC ACC AT-3’, R 5’- GCA ACT AAG TCA TAG TCC GC-3’.

Fold changes of *BAX*, *BCL-2*, and *TP53* expression were attained after normalization with β-actin gene as an endogenous control, according to the comparative 2^−ΔΔCt^ method (Livak and Schmittgen 2001).

#### *miR-155  miRmir-155*

Using samples from control and treated cells harvested at 24, 48, and 72 h, a starting amount of 500 ng of extracted miRNA, was reverse transcribed into cDNA using the miRCURY LNA RT Kit (Qiagen, Germany) in T-100 thermal cycler (Bio-Rad, CA, USA), according to the manufacturer’s instructions. RT-qPCR analyses for *miR-155 * were performed using the miRCURY LNA SYBR® Green PCR Kit (Qiagen, Germany) in a StepOnePlus Real-Time PCR System (Applied Biosystems, Thermo Fischer Scientific, USA). The cycling conditions were as follows: initial activation at 95°C for 2 min, followed by 40 cycles of 95°C for 10 s, 56°C for 60s. The small nuclear RNA (*U6*) was used as an endogenous control for data normalization and calculation of the relative expression according to the comparative threshold cycle method 2^−ΔΔCT^ (Livak and Schmittgen 2001). Specific miRCURY LNA miRNA PCR assays (Qiagen, Germany) for *miR-155 *and U6 were used (Hukowska-Szematowicz et al. 2023).

All qPCR reactions were conducted in triplicate for each sample, and data analysis was performed using average values. Melting curve analyses were performed after qPCR to prove amplification of the specific products of interest. Non-template controls were included within each run to ensure the absence of non-specific amplification for the genes used, throughout the entire study.

### DNA extraction and global methylation of DNA

Analysis of 5-methyl cytosine (5-mC) %, was implemented to measure the methylated DNA fraction of WI-38 cell line after OTA treatment. DNA samples were extracted from OTA-treated WI-38 cells (22.5 μM, 11.25 μM, and 5.6 μM), as well as from untreated control cells at different time intervals (24, 48, and 72 h). The QIAamp DNA Mini kit (Qiagen, Hilden, Germany) was used, following the instructions of the manufacturers. The extracted DNA purity and concentration were determined by 260/280 nm absorbance ratio, using NanoDrop One spectrophotometer (Thermo Fisher Scientific, MA, USA).

A concentration of 100 ng of extracted DNA was used for absolute quantification of 5-mC, using MethylFlash Methylated DNA Quantification Kit (Epigentek, NY, USA) as indicated by the manufacturer. A standard curve was generated using the positive control within the kit. The standard curve slope was calculated, and optical density (OD) was determined by reading the absorbance at 450 nm using Infinite F50 microplate reader (Tecan, Switzerland). The 5-mC concentration was determined in each sample according to this equation: 5-mC% = [(sample OD − negative control OD)/(slope * DNA quantity)] × 100. The reactions were performed in triplicates and average values were used for statistical analysis of data (Okuda et al. 2023).

### Statistical analysis

Continuous data were presented as mean and standard deviation (SD). To determine the significance of differences among different concentrations of toxin over time, Two-way repeated measures analysis of variance (ANOVA) followed by Bonferroni’s adjusted multiple pairwise comparisons were used. ANOVA test residuals were assessed for normality using Q–Q plots and for homogeneity. Ordinary one-way ANOVA followed by Bonferroni’s corrected multiple pairwise comparisons between different concentrations or Welch’s ANOVA followed by Dunnett’s T3 multiple pairwise comparisons between different concentrations were used to assess the significance of difference in experiments involving one independent factor. Spearman correlation coefficient was calculated to assess the pairwise association between different parameters. The analyses were performed using the GraphPad Prism version 9.4.0 for MacOS, GraphPad Software (San Diego, CA, USA). Statistical significance was assumed at *p* values < 0.05.

## Results and discussion

**Fig. 2:**
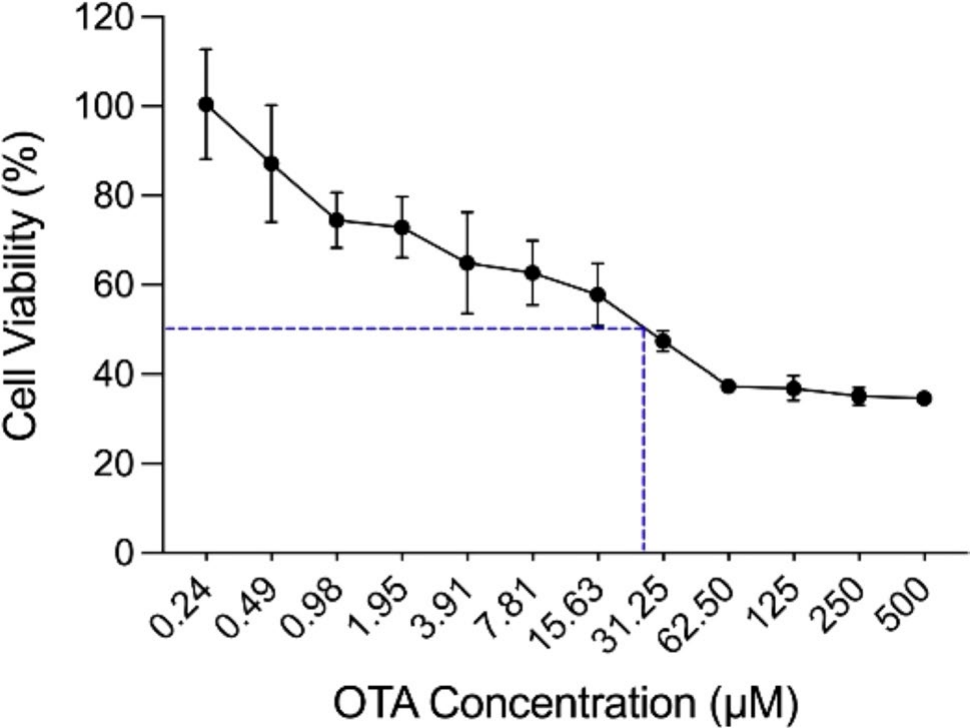
Cytotoxic effects and IC50 of OTA on WI-38 cells. Exponentially growing cells were treated with OTA at different concentrations ranging from 0.24 to 500 μM for 48 h. Cell viability was evaluated by MTT assay and expressed as percentages of control. Results are presented as mean ± SD of three independent experiments

OTA cytotoxicity was assessed using MTT assay with different OTA concentrations. The results revealed a marked OTA-induced reduction of the viable cells, in a dose-dependent pattern, with an IC50 identified at OTA concentration of 22.38 μM (Fig. 2).

**Fig. 3:**
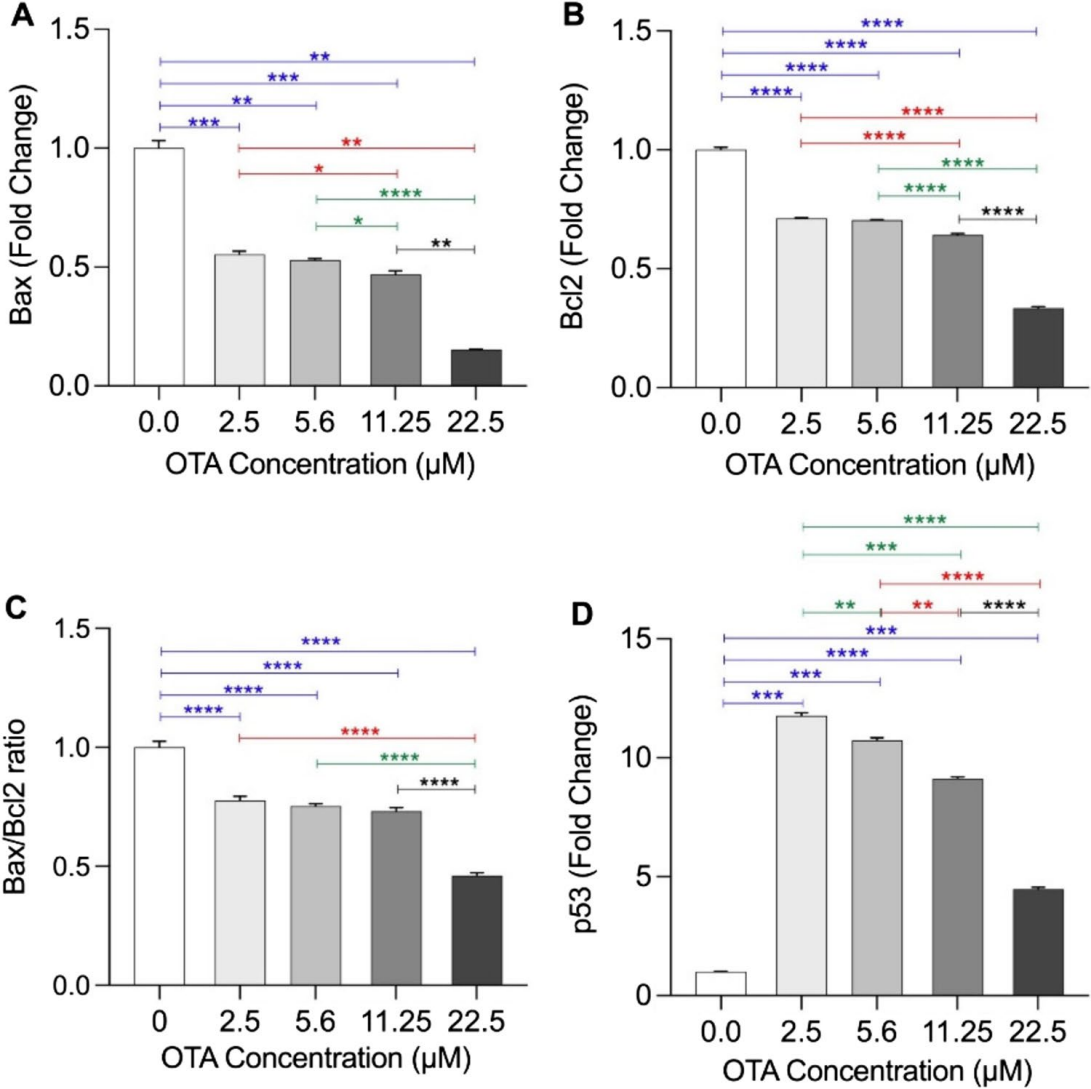
Fold changes of *BAX*, *BCL-2* and *TP53* mRNA levels. WI-38 cells were treated with 2.5 μM, 5.6 μM, 11.25 μm, and 22.5 μM concentrations of OTA for 48 h. **A ***BAX *fold changes, **B** *BCL-2* fold changes, **C** *BAX* /*BCL-2* ratio, and **D** *TP53* fold changes in test cells relative to the controls, after normalization with β-actin. Data are presented as mean ± SD of triplicate samples with the statistically significant differences indicated by p* < 0.05, p** < 0.01, p*** < 0.001, and p**** < 0.0001

*BAX* and *BCL-2* mRNA expression levels were downregulated. Likewise, *BAX*/*BCL-2* ratio declined in a dose-dependent pattern with the least ratio detected at the highest OTA concentration (22.5 μM). Contrariwise, *TP53* mRNA expression levels were upregulated at all OTA concentrations. A significant dose–dependent decline in *BAX*, *BCL-2*, and* TP53* fold changes was observed, with the least ones identified at the highest OTA concentration (22.5 μM) (Fig. 3).

**Fig. 4:**
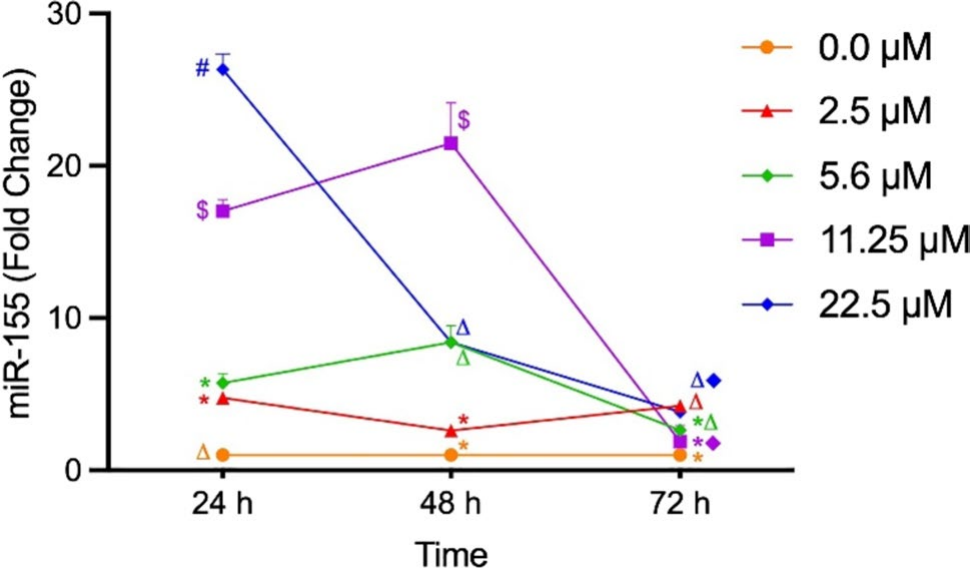
Altered *miR-155 *fold changes at different OTA concentrations. Fold changes of *miR-155 *in OTA-treated cells relative to the control monitored after 24, 48, and 72 h of OTA exposure. Fold changes were calculated after normalization with U6 and presented as mean ± SD of triplicate samples. Results having the same symbol are not significantly different (adjusted *p* > 0.05) while those having different symbols are significantly different (adjusted *p* < 0.05)

Upregulation of the *miR-155* expression at all OTA concentrations throughout the 3 days of the study, with the highest fold change demonstrated at the highest concentration (22.5 μM). However, *miR-155* fold changes showed significant reduction over time at OTA concentrations; 22.5 μM, 11.25 μM, and 5.6 μM (Fig. 4).

**Fig. 5:**
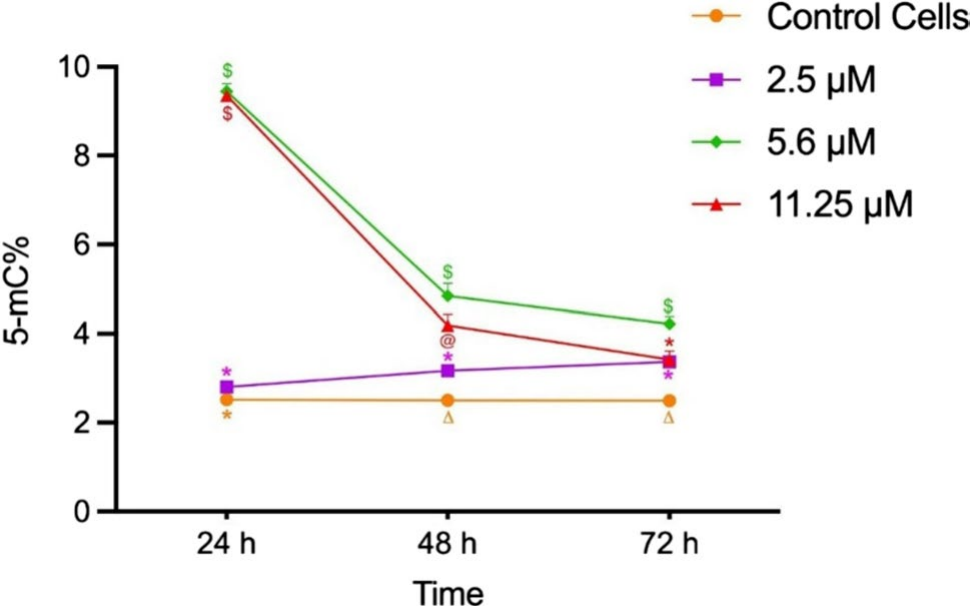
Global DNA methylation pattern in WI-38 cells after exposure to OTA. Results were recorded after exposure to subtoxic OTA concentrations (2.5 μM, 5.6 μM, and 11.25 μm) over three successive days. Data of 5-methylcytosine (5-mC%) percentage are presented as mean (SD) of triplicate samples. Results presented with the same symbol are not significantly different (adjusted *p* > 0.05), while those having different symbols are significantly different (adjusted *p* < 0.05)

**Fig. 6:**
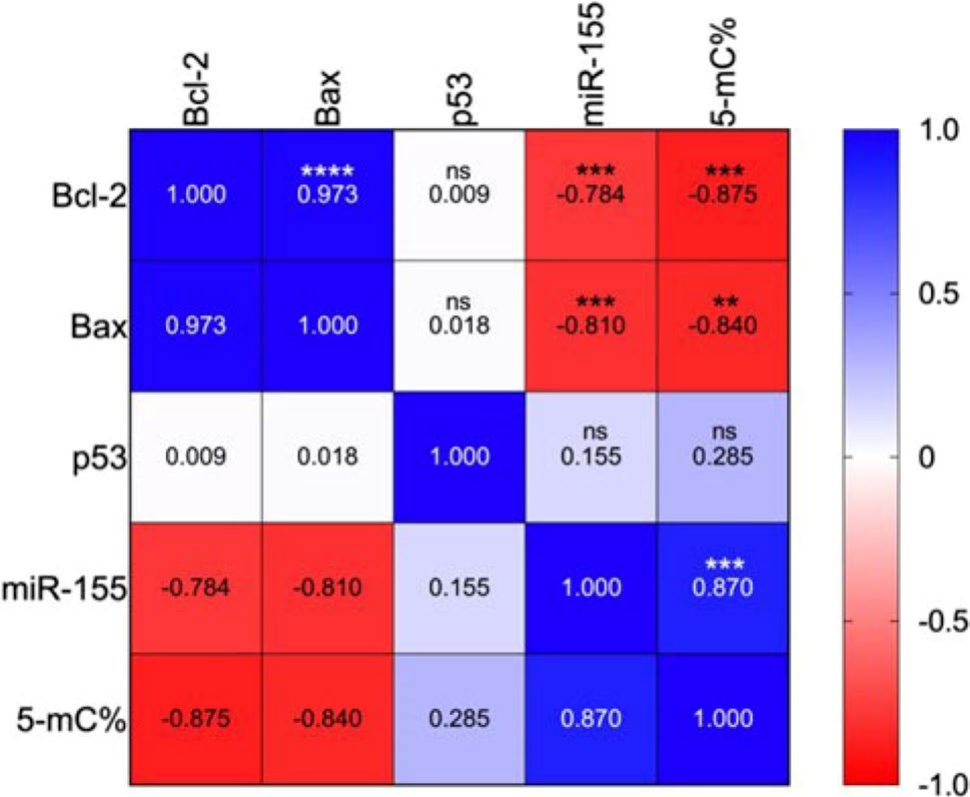
Heatmap of the correlation matrix of BCL-2, BAX, TP53, *miR-155 *fold changes, and 5-methylcytosine percentage after 48 h of exposure to OTA. Numbers are Spearman correlation coefficients for 12–15 samples. **p < 0.01, ***p < 0.001, ****p < 0.0001, ns: non-significant

Elevated 5-methylcytosine percentage (5-mC%) indicates global DNA hypermethylation, at various OTA concentrations with differential changes over time (Fig. 5). *BCL-2* fold changes were negatively correlated with *miR-155* fold change and 5mC%. Another significant negative correlation was identified between *BAX* fold change from one side with *miR-155* fold change and 5mC% on the other side. However, significant positive correlations were detected between *BAX* and *BCL-twofold* changes, besides *miR-155* fold change and 5mC% (Fig. 6).

OTA is one of the commonest environmental pollutants that may implicate human lung cells. Our records indicate the sensitivity of WI-38 cells to the effect of OTA with an IC50 detected at 22.38 μM. In 2016, Abassi et al. studied the effects of different mycotoxins including OTA on fetal lung fibroblast-like cells transfected with MYC (MRC5-myc) and reported the IC50 of OTA at 20 μM, which is agreed with our finding (Abassi et al. 2016).

Several explanations were proposed to describe OTA-induced toxicity such as DNA damage that activates P53 protein and subsequently activates mitochondrial pathway of cell death (Bouaziz et al. 2008). Another proposed mechanism is the OTA mutagenic effect that generates genotoxic metabolites (Obrecht-Pflumio et al. 1999; Obrecht-Pflumio and Dirheimer 2000). In actuality, the fundamental mechanisms of OTA-induced mutation and tumorigenicity are still indistinct. To further assess the effects of OTA-induced genotoxicity, and oncogenic predisposition in WI-38 cells, mRNA expression of apoptosis regulatory genes; *BAX* and *BCL-2* was assessed and found to be significantly reduced with increasing OTA concentration. The *BAX*/*BCL-2* ratio was also reduced showing the same dose-dependent decline trend. In lung cancer, the relationship between pro- and anti-apoptotic proteins is ambiguous and debatable (Porebska et al. 2006). Changes in *TP53* gene expression were also monitored in this study, and *TP53* mRNA showed a significantly increased fold change after treatment with different OTA concentrations. Notably, *TP53* overexpression has been significantly decreased with increasing toxin concentration, with the least level detected at the highest OTA dose (22.5 μM).

It is well known that neoplastic cells must circumvent the tumor suppressor genes’ primary role in regulating cell proliferation. Though activated *TP53* can initiate apoptosis or just stop the cell-cycle progression until the stress subsides to normal, the numerous effects of activated *TP53 *are intricate and context-dependent. They vary according to the cell type, stress severity, persistent exposure of the cell to stress, and DNA damage (Hanahan and Weinberg 2011). Obviously, the primary function of P53 in apoptosis is via regulating the *BCL-2* family activity (Hemann and Lowe 2006), which is dysregulated in response to OTA exposure (Bouaziz et al. 2011). It has been previously reported that, *BCL-2 *family proteins are the principle regulators of the intrinsic apoptotic pathway, which is the physiologically dominant pathway of cell death, and cells with low Bax protein levels are designated as apoptosis resistant cells (Cotter 2009; Singh et al. 2019). Activated P53 is known to upregulate *BAX* gene expression, which in turn overcomes the *BCL-2 *anti-apoptotic effects, and accordingly, *BAX* deficient cells are refractory to the stimuli that initiate P53-dependent apoptosis. In other words, deregulated expression of *BCL-2* promotes P53-dependent apoptosis that is diminished in *BAX* deficient cells (McCurrach et al. 1997; Yin et al. 1997). Hence, the P53 impact the fate of the cells, in response to various stress conditions, by regulating the *BAX*/*BCL-2*-ratio (Hemann and Lowe 2006). This could be a possible explanation of our results, where we can infer that the reduced expression of *BAX* and the deregulated *BCL-2* expression together with low *BAX*/*BCL-2* ratio resulted in attenuated P53-mediated apoptosis after exposure of WI-38 cells to OTA. Consequently, the WI-38 cells exposed to OTA were possibly able to evade the tumor suppressor effects of *TP53* gene and become more prone to the oncogenic effects of the *BCL-2* family. This would eventually promote the likelihood of cellular malignant transformation. The *BAX* gene upstream regulator is the *TP53* gene product (Miyashita and Reed 1995). Nevertheless, the *BAX* promoter is known to be activated by the wild type of *TP53*, but not the mutant type. Hence, the *TP53* expression and its mutational status could be linked to the changes in *BAX* gene expression. A study on melanoma cells had shown that cells with low *BAX*/*BCL-2* ratio (< 1) are resistant to apoptosis and cells with high *BAX*/*BCL-2* are sensitive to apoptosis. Thereby, it was inferred that low *BAX* levels in these malignant cells might be due to *TP53* mutation (Raisova et al. 2001). This finding can be proposed as another possible elucidation to the results of our study where the deregulated expression of the apoptosis regulatory genes; low *BAX* fold changes and the overexpression of *TP53* may be an indicator of presence of mutant *TP53* after exposure of the WI-38 cells to OTA. It can also support the notion that the dose-dependent marked reduction in *BAX*/*BCL-2* ratio may be a marker of OTA-induced carcinogenicity on WI-38 cells. Our findings in this aspect are consistent with earlier research that found deregulated *BCL-2* expression to be a feature of many human cancers (Singh et al. 2015). In particular, low expression of *BCL-2*, was observed in lung cancer (Porebska et al. 2006).

The current results denote a lack of correlation between *TP53* and *BCL-2* or *BAX* mRNA levels in OTA-treated WI-38 cells. This may refer to the possibility of disrupted normal regulatory pathway between *TP53* and *BCL-2* or *BAX* in OTA-treated WI-38 cells. Our results in this regard are in concordance with the results of a previous study that detected P53 protein in lung cancer tissue and its absence in the normal bronchial tissue. Interestingly, *BCL-2 *protein was identified in some cancer cells while absent in others within the same lung cancer specimen. The same study also found a statistically insignificant relationship between the apoptotic markers; *TP53* and *BCL-2* or *BAX* (Porebska et al. 2006), a finding that was also supported by another study reporting the lack of correlation between P53 and *BCL-2 *(Apolinario et al. 1997). We have also recognized the presence of a positive correlation between *BCL-2* and *BAX*. Opposite to our findings, an earlier study reported a negative correlation between *BCL-2* and *BAX* immuno-reactivity in lung cancer (Gregorc et al. 2003).

To our knowledge, our current study is the first to document *mir-155 *upregulation in WI-38 cells after exposure to different OTA concentrations. Such upregulation is characterized by being dynamic as it increased with higher OTA concentrations and decreased gradually over time. *miRr-155* is predominantly known for its oncogenic roles in lung cancer and several researches established a link between the overexpression of *miR-155* and lung cancer diagnosis, prognosis and pathogenesis (Higgs and Slack 2013, Hou et al. 2016; Shao et al. 2019). The elevated *miR-155* expression in this study is supported by earlier reports suggesting that upregulation of *miR-155* could activate *TP53* and create a positive feedback loop. In turn, *TP53* controls the transcription of *miR-155*, which regulates the cell cycle, cell proliferation, differentiation, and apoptosis (Wang et al. 2018). Interestingly, a previous study had strongly linked *miR-155* expression to the presence of *TP53* mutants (Madrigal et al. 2021), thus providing another possible explanation to our results that should be kept in mind for further genotypic investigations. Importantly, Gu and colleagues (2017) reported that *miR-155* downregulates *BAX*, thus efficiently lowers the *BAX/BCL-2* ratio in human lung adenocarcinoma A549R cell line. This goes in line with our above-mentioned result regarding reduction of *BAX/BCL-2* ratio in response to increasing OTA concentration. Taken together, we can infer from the current findings that exposure to OTA might upregulate oncomiR-155 and TP53 but downregulate *BAX *gene and lower *BAX/BCL-2*-ratio, a state that favors carcinogenesis due to inhibition of apoptosis which represents the hallmark of our study hypothesis. Inhibition of apoptosis, resisting cell death, and evading growth suppressors (such as TP53) are crucial steps in the development of carcinogenesis in various human malignancies (Hanahan and Weinberg 2011).

Epigenetic alterations in particular, DNA methylation and miRNAs, are regarded as the most prevalent genomic abnormalities during development, spread, and progression of cancer (Wang et al. 2017). In the field of investigating OTA toxicity, DNA methylation has received a lot of attention, and former studies had reported the ability of some environmental contaminants to alter the DNA methylation pattern (Brocato and Costa 2013; Lambrou et al. 2012; Niedzwiecki et al. 2013). Despite having several studies on methylation of individual gene promoters, a few research studies were conducted to examine the genome-wide methylation activity of lung cancer cells. The alteration of the methylation profile due to long term exposure to OTA was also noticed in a study done by Li et al. (2015) whose results supported that only detecting the global DNA methylation is sufficient to explore the role of DNA methylation in OTA-induced nephrotoxicity. Our study on fetal lung fibroblasts cell line demonstrated a significant increase in the global DNA methylation with all three subtoxic OTA concentrations, as compared to the control cells. The observed global DNA hypermethylation displayed a dose-dependent increasing trend with increasing toxin concentration; then, a significant decline started to appear at the highest concentration 11.25 μM. Interestingly, we recorded two different patterns of changes in global DNA methylation, according to the toxin concentration. At low OTA concentration (2.5 μM), the hypermethylation was significantly increasing over time; however at higher toxin concentrations (5.6 μM and 11.25 μm), it was significantly decreasing with time. This could raise concerns about the dangers of chronic exposure to low dosages of OTA. Our observations are in accordance with those of Chen et al. (2021), who compared global methylation level between the individual cell clusters in lung adenocarcinoma, and declared it as a fundamental event toward the progression of lung adenocarcinoma especially invasion and metastasis. On the other hand, exposure to OTA yielded either global DNA hypomethylation, as detected in HepG2 and PK15 cells (Zheng et al. 2013; Zhou et al. 2017), or did not cause any change in the global methylation status as in MDCK and BME-UV1 cells [65]. Such discrepancies might be attributed to the different cell lines used and different tissue-specific responses to OTA. Consequently, it might be suggested that the identified hypermethylation in WI-38 cells may provide an early sign of developing cell transformation after exposure to subtoxic concentrations of OTA for longer time.

miRNAs can regulate DNA methylation. In human malignancies, a reciprocal regulatory loop has been observed between miRNAs and DNA methylation (Li 2015; Wang et al. 2017). In the current study, a positive correlation was detected between *oncomiR-155* and 5mC %. The dynamic and the dose-dependent changes of onco*miR-155* fold changes and global DNA methylation were parallel during OTA-induced toxicity in WI-38 cells. A recent publication in 2023 denoted that the two regulatory mechanisms, DNA methylation and miRNAs expression, can impact specific biological processes, such as apoptosis, metastasis, and cell proliferation. The crosstalk and the dysregulation between DNA methylation and miRNAs are suggestive of developing human cancers (Saviana et al. 2023). Additionally, we identified a negative correlation between the apoptotic regulatory genes (*BAX* and *BCL-2*) and 5mC%, which favors inhibition of apoptosis that could be considered as a potentially useful indicator for the possibility of malignant transformation due to OTA exposure. This may open a window to discover a new target for detecting OTA-induced pathogenicity in the lung tissue. Probably these changes may suggest that long-term OTA exposure could disturb the oncogenic *miR-155* and the DNA methylation profile, which might be a possible mechanism of OTA-induced cytotoxicity and possible carcinogenicity in WI-38 cells. This is supported by the previously mentioned study of Li et al. (2015), who related the dose-dependent and dynamic changes of the DNA global methylation to OTA-induced nephrotoxicity and to the increased expression of DNA methyltransferase.

Despite the numerous described hypotheses, the role of different molecular and epigenetic mechanisms of OTA-induced lung toxicity and carcinogenicity are only partially understood. Our study is an initial step to comprehend the effects of OTA on the molecular and cellular interactions via altering the expression of some epigenetic markers, specifically global DNA methylation and *oncomiR-155*. Our findings aimed to improve the efforts to avoid or minimize its toxicity at the molecular and cellular levels. However, further in-depth research is required to explore the epigenetic and molecular pathways of OTA-induced lung toxicity and carcinogenicity.

One of the limitations of our study was to identify the *TP53* genotype to relate it to the increased expression of *oncomiR-155* as well as studying the methylation pattern of the promoters of the identified genes. Using animal models to elucidate the in vivo effects of OTA on the lungs would also be another area that needs further exploration.

## Conclusions

Under the present experimental conditions, the current results present a potential pathway of OTA-induced cytotoxicity and possible carcinogenicity in WI-38 cells. *TP53* is upregulated in response to OTA-mediated DNA damage. The activated *TP53* gene may also promote the induction of the oncomir *miR-155* which in turn, downregulates *BAX* mRNA level, thus resulting in reduction of the *BAX*/*BCL-2* ratio and inhibition of *TP53*-mediated apoptosis. Of note, *miR-155* was positively correlated to global DNA hypermethylation in OTA-treated WI-38 cells, a result that might be regarded as an early epigenetic response for OTA-induced toxicity in WI-38 cells. Therefore, there is an urgent need to update the estimated human tolerable dosages of OTA in order to give a reliable basis for evaluating the associated health risks that jeopardize the general population.

## Data Availability

All data generated or analyzed during this study are included in the research article.

## References

[CR1] Abassi H, Ayed-Boussema I, Shirley S, Abid S, Bacha H (2016). Ochratoxin A and T-2 toxin induce clonogenicity and cell migration in human colon carcinoma and fetal lung fibroblast cell lines. J Biochem Mol Toxicol.

[CR2] Apolinario RM, van der Valk P, de Jong JS, Deville W, van Ark-Otte J, Dingemans AM, van Mourik JC, Postmus PE, Pinedo HM, Giaccone G (1997). Prognostic value of the expression of p53, bcl-2, and bax oncoproteins, and neovascularization in patients with radically resected non-small-cell lung cancer. J Clin Oncol.

[CR3] Awuchi CG, Ondari EN, Ogbonna CU, Upadhyay AK, Baran K, Okpala COR, Korzeniowska M, Guiné RPF (2021): Mycotoxins affecting animals, foods, humans, and plants: types, occurrence, toxicities, action mechanisms, prevention, and detoxification strategies—a revisit. 10, 1279 10.3390/foods10061279 PMC8228748 34205122

[CR4] Beasley AB, Chen FK, Isaacs TW, Gray ES (2022). Future perspectives of uveal melanoma blood based biomarkers. Br J Cancer.

[CR5] Bouaziz C, Sharaf El Dein O, El Golli E, Abid-Essefi S, Brenner C, Lemaire C, Bacha H (2008). Different apoptotic pathways induced by zearalenone, T-2 toxin and ochratoxin A in human hepatoma cells. Toxicology.

[CR6] Bouaziz C, Sharaf el dein O, Martel C, El Golli E, Abid-Essefi S, Brenner C, Lemaire C, Bacha H (2011): Molecular events involved in ochratoxin A induced mitochondrial pathway of apoptosis, modulation by Bcl-2 family members. Environ Toxicol 26, 579-90 10.1002/tox.20581 20549612

[CR7] Brocato J, Costa M (2013). Basic mechanics of DNA methylation and the unique landscape of the DNA methylome in metal-induced carcinogenesis. Crit Rev Toxicol.

[CR8] Chain EPanel oCitF et al. (2020): Risk assessment of ochratoxin A in food. 18, e06113 10.2903/j.efsa.2020.6113 PMC10464718 37649524

[CR9] Chen Q-F, Gao H, Pan Q-Y, Wang Y-J, Zhong X-N (2021). Analysis at the single-cell level indicates an important role of heterogeneous global DNA methylation status on the progression of lung adenocarcinoma. Sci Rep.

[CR10] Costa JG, Saraiva N, Guerreiro PS, Louro H, Silva MJ, Miranda JP, Castro M, Batinic-Haberle I, Fernandes AS, Oliveira NG (2016). Ochratoxin A-induced cytotoxicity, genotoxicity and reactive oxygen species in kidney cells: an integrative approach of complementary endpoints. Food Chem Toxicol.

[CR11] Cotter TG (2009). Apoptosis and cancer: the genesis of a research field. Nat Rev Cancer.

[CR12] Ellinger-Ziegelbauer H, Stuart B, Wahle B, Bomann W, Ahr HJ (2004). Characteristic expression profiles induced by genotoxic carcinogens in rat liver. Toxicol Sci.

[CR13] Gironella M (2007). Tumor protein 53-induced nuclear protein 1 expression is repressed by miR-155, and its restoration inhibits pancreatic tumor development. Proc Natl Acad Sci U S A.

[CR14] Gregorc V, Ludovini V, Pistola L, Darwish S, Floriani I, Bellezza G, Sidoni A, Cavaliere A, Scheibel M, De Angelis V, Bucciarelli E, Tonato M (2003). Relevance of p53, bcl-2 and Rb expression on resistance to cisplatin-based chemotherapy in advanced non-small cell lung cancer. Lung Cancer.

[CR15] Gu S, Lai Y, Chen H, Liu Y, Zhang Z (2017). miR-155 mediates arsenic trioxide resistance by activating Nrf2 and suppressing apoptosis in lung cancer cells. Sci Rep.

[CR16] Hanahan D, Weinberg RA (2011). Hallmarks of cancer: the next generation. Cell.

[CR17] Hemann MT, Lowe SW (2006). The p53-Bcl-2 connection. Cell Death Differ.

[CR18] Higgs G, Slack F (2013). The multiple roles of microRNA-155 in oncogenesis. J Clin Bioinforma.

[CR19] Hou Y, Wang J, Wang X, Shi S, Wang W, Chen Z (2016). Appraising microRNA-155 as a noninvasive diagnostic biomarker for cancer detection: a meta-analysis. Medicine (baltimore).

[CR20] Hukowska-Szematowicz B, Ostrycharz E, Dudzińska W, Roszkowska P, Siennicka A, Wojciechowska-Koszko I (2023) Digital PCR (dPCR) quantification of miR-155–5p as a potential candidate for a tissue biomarker of inflammation in rabbits infected with lagovirus europaeus/rabbit hemorrhagic disease virus (RHDV). Viruses 15 10.3390/v15071578 PMC10386091 37515264

[CR21] Jakšić D, Sertić M, Kocsubé S, Kovačević I, Kifer D, Mornar A, Nigović B, Šegvić Klarić M (2020). Post-flood impacts on occurrence and distribution of mycotoxin-producing aspergilli from the sections circumdati, flavi, and nigri in indoor environment. J Fungi.

[CR22] Lambrou A, Baccarelli A, Wright RO, Weisskopf M, Bollati V, Amarasiriwardena C, Vokonas P, Schwartz J (2012). Arsenic exposure and DNA methylation among elderly men. Epidemiology.

[CR23] Lane DP, Crawford LV (1979). T antigen is bound to a host protein in SV40-transformed cells. Nature.

[CR24] Li X (2015) Dynamic changes of global DNA methylation and hypermethylation of cell adhesion-related genes in rat kidneys in response to ochratoxin A. World mycotoxin journal v. 8, pp. 465–476–2015 v.8 no.4

[CR25] Linzer DI, Levine AJ (1979). Characterization of a 54K dalton cellular SV40 tumor antigen present in SV40-transformed cells and uninfected embryonal carcinoma cells. Cell.

[CR26] Liu F, Song D, Wu Y, Liu X, Zhu J, Tang Y (2017). MiR-155 inhibits proliferation and invasion by directly targeting PDCD4 in non-small cell lung cancer. Thorac Cancer.

[CR27] Livak KJ, Schmittgen TD (2001). Analysis of relative gene expression data using real-time quantitative PCR and the 2(-Delta Delta C(T)) Method. Methods.

[CR28] Madrigal T, Hernández-Monge J, Herrera LA, González-De la Rosa CH, Domínguez-Gómez G, Candelaria M, Luna-Maldonado F, Calderón González KG, Díaz-Chávez J (2021). Regulation of miRNAs expression by mutant p53 gain of function in cancer. Front Cell Dev Biol.

[CR29] Marin-Kuan M, Ehrlich V, Delatour T, Cavin C, Schilter B (2011). Evidence for a role of oxidative stress in the carcinogenicity of ochratoxin a. J Toxicol.

[CR30] McCurrach ME, Connor TM, Knudson CM, Korsmeyer SJ, Lowe SW (1997). bax-deficiency promotes drug resistance and oncogenic transformation by attenuating p53-dependent apoptosis. Proc Natl Acad Sci U S A.

[CR31] Miyashita T, Reed JC (1995). Tumor suppressor p53 is a direct transcriptional activator of the human bax gene. Cell.

[CR32] Ng CT, Dheen ST, Yip WC, Ong CN, Bay BH, Lanry Yung LY (2011). The induction of epigenetic regulation of PROS1 gene in lung fibroblasts by gold nanoparticles and implications for potential lung injury. Biomaterials.

[CR33] Niedzwiecki MM, Hall MN, Liu X, Oka J, Harper KN, Slavkovich V, Ilievski V, Levy D, van Geen A, Mey JL, Alam S, Siddique AB, Parvez F, Graziano JH, Gamble MV (2013). A dose-response study of arsenic exposure and global methylation of peripheral blood mononuclear cell DNA in Bangladeshi adults. Environ Health Perspect.

[CR34] Obrecht-Pflumio S, Chassat T, Dirheimer G, Marzin D (1999). Genotoxicity of ochratoxin A by Salmonella mutagenicity test after bioactivation by mouse kidney microsomes. Mutat Res.

[CR35] Obrecht-Pflumio S, Dirheimer G (2000). In vitro DNA and dGMP adducts formation caused by ochratoxin A. Chem Biol Interact.

[CR36] Okuda K (2023). Pivotal role for S-nitrosylation of DNA methyltransferase 3B in epigenetic regulation of tumorigenesis. Nat Commun.

[CR37] Porebska I, Wyrodek E, Kosacka M, Adamiak J, Jankowska R, Harłozińska-Szmyrka A (2006). Apoptotic markers p53, Bcl-2 and Bax in primary lung cancer. In Vivo.

[CR38] Raisova M, Hossini AM, Eberle J, Riebeling C, Wieder T, Sturm I, Daniel PT, Orfanos CE, Geilen CC (2001). The Bax/Bcl-2 ratio determines the susceptibility of human melanoma cells to CD95/Fas-mediated apoptosis. J Invest Dermatol.

[CR39] Ramadan MA, Shawkey AE, Rabeh MA, Abdellatif AO (2019). Expression of P53, BAX, and BCL-2 in human malignant melanoma and squamous cell carcinoma cells after tea tree oil treatment in vitro. Cytotechnology.

[CR40] Rhee KH, Yang SA, Pyo MC, Lim J-M, Lee K-W (2023) MiR-155–5p Elevated by ochratoxin A induces intestinal fibrosis and epithelial-to-mesenchymal transition through TGF-&beta; regulated signaling pathway in vitro and in vivo. 15, 473 10.3390/toxins15070473 PMC10467050 37505742

[CR41] Said YM, El-Gamel NEA, Ali SA, Mohamed AF (2022). Evaluation of human Wharton’s jelly-derived mesenchymal stem cells conditioning medium (hWJ-MSCs-CM) or scorpion venom breast cancer cell line in vitro. J Gastrointest Cancer.

[CR42] Saviana M, Le P, Micalo L, Del Valle-Morales D, Romano G, Acunzo M, Li H, Nana-Sinkam P (2023) Crosstalk between miRNAs and DNA methylation in cancer. Genes 14 10.3390/genes14051075 PMC10217889 37239435

[CR43] Shahbazi J, Lock RB, Liu T (2013) Tumor protein 53-induced nuclear protein 1 enhances p53 function and represses tumorigenesis. Frontiers in Genetics 4 10.3389/fgene.2013.00080 PMC3652520 23717325

[CR44] Shao C, Yang F, Qin Z, Jing X, Shu Y, Shen H (2019). The value of miR-155 as a biomarker for the diagnosis and prognosis of lung cancer: a systematic review with meta-analysis. BMC Cancer.

[CR45] Singh L, Pushker N, Saini N, Sen S, Sharma A, Bakhshi S, Chawla B, Kashyap S (2015). Expression of pro-apoptotic Bax and anti-apoptotic Bcl-2 proteins in human retinoblastoma. Clin Exp Ophthalmol.

[CR46] Singh R, Letai A, Sarosiek K (2019). Regulation of apoptosis in health and disease: the balancing act of BCL-2 family proteins. Nat Rev Mol Cell Biol.

[CR47] van Delft JH, van Agen E, van Breda SG, Herwijnen MH, Staal YC, Kleinjans JC (2004). Discrimination of genotoxic from non-genotoxic carcinogens by gene expression profiling. Carcinogenesis.

[CR48] van der Merwe KJ, Steyn PS, Fourie L, Scott DB, Theron JJ (1965). Ochratoxin A, a toxic metabolite produced by Aspergillus ochraceus Wilh. Nature.

[CR49] Wang S, Wu W, Claret FX (2017). Mutual regulation of microRNAs and DNA methylation in human cancers. Epigenetics.

[CR50] Wang Y, Zheng Z-j, Jia Y-j, Yang Y-l, Xue Y-m (2018). Role of p53/miR-155-5p/sirt1 loop in renal tubular injury of diabetic kidney disease. J Transl Med.

[CR51] Warensjö Lemming E, Montano Montes A, Schmidt J, Cramer B, Humpf HU, Moraeus L, Olsen M (2020). Mycotoxins in blood and urine of Swedish adolescents-possible associations to food intake and other background characteristics. Mycotoxin Res.

[CR52] Yang SA, Rhee KH, Yoo HJ, Pyo MC, Lee KW (2023). Ochratoxin A induces endoplasmic reticulum stress and fibrosis in the kidney via the HIF-1α/miR-155-5p link. Toxicol Rep.

[CR53] Yin C, Knudson CM, Korsmeyer SJ, Van Dyke T (1997). Bax suppresses tumorigenesis and stimulates apoptosis in vivo. Nature.

[CR54] Yu C-Y, Li T-C, Wu Y-Y, Yeh C-H, Chiang W, Chuang C-Y, Kuo H-C (2017). The circular RNA circBIRC6 participates in the molecular circuitry controlling human pluripotency. Nat Commun.

[CR55] Zerdoumi Y, Kasper E, Soubigou F, Adriouch S, Bougeard G, Frebourg T, Flaman JM (2015). A new genotoxicity assay based on p53 target gene induction. Mutat Res Genet Toxicol Environ Mutagen.

[CR56] Zhang J, Cheng C, Yuan X, He JT, Pan QH, Sun FY (2014). microRNA-155 acts as an oncogene by targeting the tumor protein 53-induced nuclear protein 1 in esophageal squamous cell carcinoma. Int J Clin Exp Pathol.

[CR57] Zheng J, Zhang Y, Xu W, Luo Y, Hao J, Shen XL, Yang X, Li X, Huang K (2013). Zinc protects HepG2 cells against the oxidative damage and DNA damage induced by ochratoxin A. Toxicol Appl Pharmacol.

[CR58] Zhou Y, Gan F, Hou L, Zhou X, Adam Ibrahim YA, Huang K (2017). Modulations of DNMT1 and HDAC1 are involved in the OTA-induced cytotoxicity and apoptosis in vitro. Chem Biol Interact.

[CR59] Zhu HZ, Hou J, Guo Y, Liu X, Jiang FL, Chen GP, Pang XF, Sun JG, Chen ZT (2018). Identification and imaging of miR-155 in the early screening of lung cancer by targeted delivery of octreotide-conjugated chitosan-molecular beacon nanoparticles. Drug Deliv.

[CR60] Zhu L, Zhang B, Dai Y, Li H, Xu W (2017) A review: epigenetic mechanism in ochratoxin A toxicity studies. Toxins (Basel) 9 10.3390/toxins9040113 PMC5408187 28333080

